# Matching Biomedical Ontologies *via* a Hybrid Graph Attention Network

**DOI:** 10.3389/fgene.2022.893409

**Published:** 2022-07-22

**Authors:** Peng Wang, Yunyan Hu

**Affiliations:** ^1^ School of Computer Science and Engineering, Southeast University, Nanjing, China; ^2^ School of Cyber Science and Engineering, Southeast University, Nanjing, China

**Keywords:** biomedical ontology, ontology matching, graph attention network, embedding, hyperbolic attention

## Abstract

Biomedical ontologies have been used extensively to formally define and organize biomedical terminologies, and these ontologies are typically manually created by biomedical experts. With more biomedical ontologies being built independently, matching them to address the problem of heterogeneity and interoperability has become a critical challenge in many biomedical applications. Existing matching methods have mostly focused on capturing features of terminological, structural, and contextual semantics in ontologies. However, these feature engineering-based techniques are not only labor-intensive but also ignore the hidden semantic relations in ontologies. In this study, we propose an alternative biomedical ontology-matching framework BioHAN via a hybrid graph attention network, and that consists of three techniques. First, we propose an effective ontology-enriching method that refines and enriches the ontologies through axioms and external resources. Subsequently, we use hyperbolic graph attention layers to encode hierarchical concepts in a unified hyperbolic space. Finally, we aggregate the features of both the direct and distant neighbors with a graph attention network. Experimental results on real-world biomedical ontologies demonstrate that BioHAN is competitive with the state-of-the-art ontology matching methods.

## 1 Introduction

**FIGURE 1:**
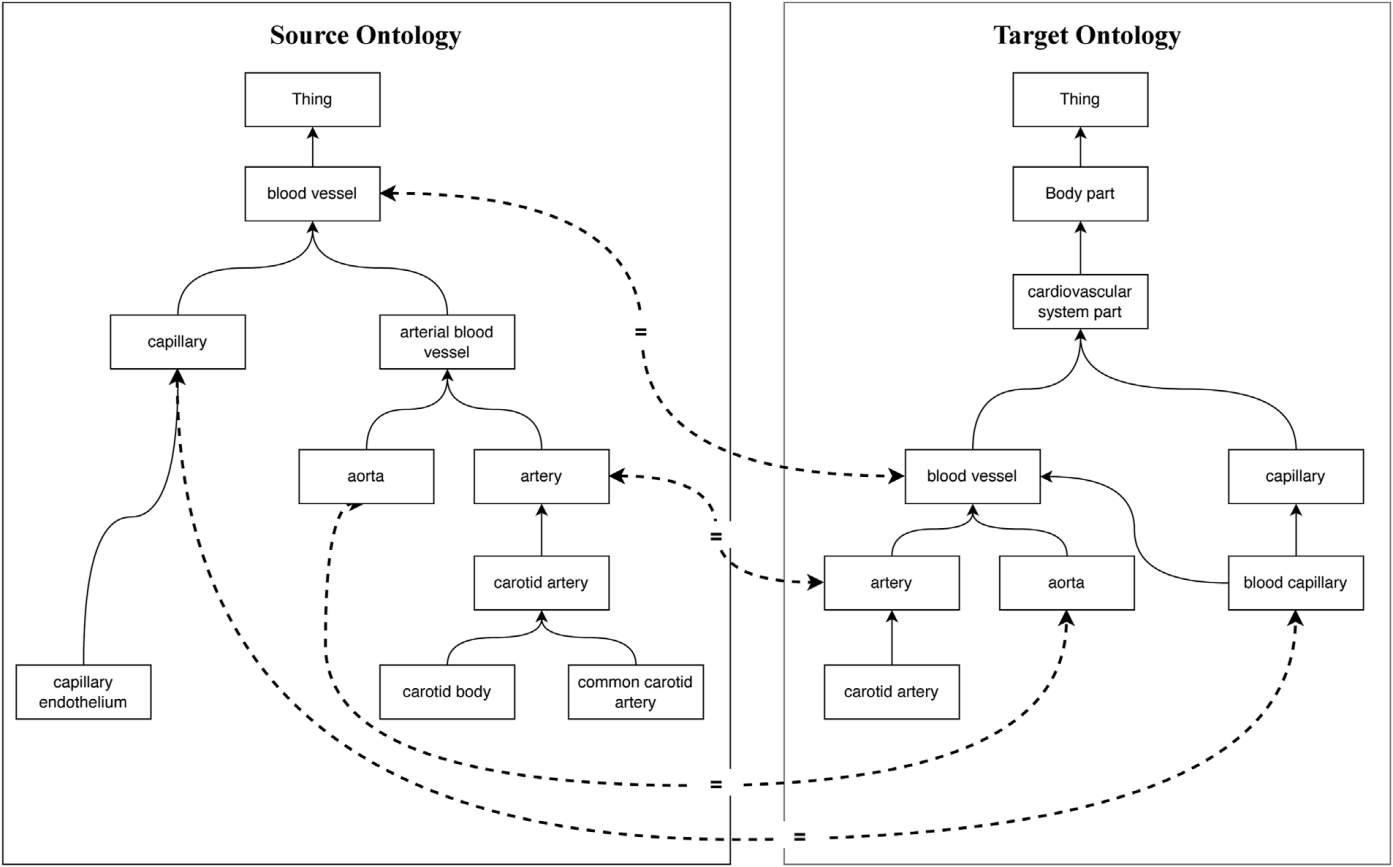
Heterogeneity of biomedical ontologies.

Ontology is an explicit, interoperable, extensible, scalable, and formal definition to describe knowledge as a set of domain vocabularies that contain concepts, relations between concepts, and individuals of concepts (Ramis et al., 2014). In past decades, various biomedical ontologies, such as the National Cancer Institute Thesaurus (NCI) (Golbeck et al., 2003), Foundation Model of Anatomy (FMA) (Rosse and Mejino, 2003), Systemized Nomenclature of Medicine (SNOMED-Clinical Terms [SNOMED-CT]) (Donnelly et al., 2006), and Uberon (Mungall et al., 2012) have been widely used for medical data format standardization (Cimino and Zhu, 2006), medical or clinical knowledge representation and integration (Isern et al., 2012), and medical decision making (De Potter et al., 2012) to provide standard semantics. With the continuous evolution of biomedical data, biomedical vocabularies have become complicated and ambiguous, which leads to challenges in developing biomedical applications. Moreover, new biomedical ontologies are constructed independently with diverse ways of defining overlapping biomedical terminologies or components, which also leads to more heterogeneity (Xie et al., 2016). As shown in Figure 1, the entities are connected *via* the *subClassOf* relation, and the equivalent concepts are linked via dotted lines. It can be found that for the same concept, “blood vessel” in the source and target ontologies, they are organized and interpreted at different levels of granularity, named conceptual heterogeneity. In addition, the concepts that share the same morphology “capillary” indicate different semantics in different ontologies, which is called semiotic heterogeneity. To implement interoperability across biomedical ontologies, discovering semantic relations between them is critically important (Xue, 2020). Ontology matching is a key technique to find semantic correspondences between the elements of different ontologies to achieve interoperability (Shvaiko and Euzenat, 2011).

Most existing ontology matching methods have focused on extracting features from terminological, structural, extensional (individuals of concepts) information, and external resources (Nezhadi et al., 2011; Otero-Cerdeira et al., 2015; Babalou et al., 2016; Chauhan et al., 2018). They use logical reasoning and rule-based techniques to extract sophisticated features, which are then used to compute the similarities of ontological elements (i.e., concepts, properties, and individuals) that promote ontology matching.

These feature-based methods (e.g., AML (Faria et al., 2013), FCA_Map (Zhao et al., 2018), LogMap (Jiménez-Ruiz and Cuenca Grau, 2011), and XMap (Djeddi and Khadir, 2014)) elaborate features of data to evaluate element similarity and derive semantic correspondences. However, the features in one ontology usually cannot be transferred to others. Consequently, the effectiveness and generality of those ontology matching methods vary significantly (Kolyvakis et al., 2018).

**FIGURE 2:**
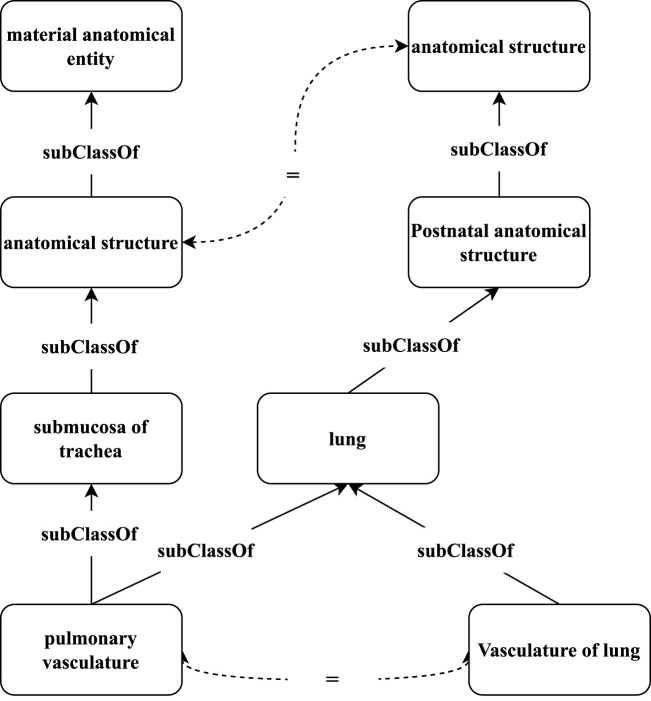
Hierarchical structure in biomedical ontologies UBERON (left) and FMA (right).

Recently, graph-based representation learning (Kipf and Welling, 2016; Hamilton et al., 2017) has become a powerful model for learning vector representations of graph-structured data. In graph neural networks (GNNs), the representation of a node is learned through recursively aggregating the representations of its local neighboring structure and propagation of features from neighboring nodes. Several studies (Chen et al., 2017; Wang Z et al., 2018; Wu et al., 2019; Sun et al., 2020) exploit GNNs for embedding-based matching in knowledge graphs (KGs), and have achieved promising results. However, existing GNN-based matching models still face some problems in ontology matching. First, ontology matching may face semantic imbalance because the distributions and amounts of semantic descriptions in different ontologies are generally different. We argue that if we can enrich the ontologies by using the metadata, given axioms, and auxiliary descriptions from external domain resources, and incorporate a rich set of semantic relationships, the derived ontologies can be matched with higher precision and recall. To overcome this problem, we consider designing an ontology-enriching method. Second, a distinguishable characteristic of biomedical ontologies, compared to open-domain knowledge bases such as YAGO (Suchanek et al., 2007), Wikidata (Vrandečić and Krötzsch, 2014), and DBpedia (Lehmann et al., 2015), is their domain specificity. These biomedical ontologies often have rich hierarchical structures that systematically organize biomedical concepts into categories and subcategories from general to specific. Figure 2 shows an example of a hierarchical structure in different biomedical ontologies. The hierarchical structures of the corresponding pairs in different ontologies are similar to some extent. For example, the hierarchy (through *subClassOf* relation) of “pulmonary vasculature” in UBERON and “Vasculature of lung” in FMA is similar, whereas the terminologies are morphologically different. Therefore, capturing such hierarchical structures would be useful for identifying aligned concepts and improving the matching performance. Finally, since different ontologies usually have heterogeneous schemas and incompleteness (Schneider and Šimkus, 2020), the matching pairs usually have some dissimilar neighboring structures. Even though we assume that the ontologies to be matched are complete, because of the schema heterogeneity, the non-isomorphism in the neighboring structures from different ontologies is still inevitable. As shown in Figure 2, the one-hop neighbors of the matching pair (“pulmonary vasculature” and “Vasculature of lung”) are different, while they share the same distant neighbor “anatomical structure.” Motivated by the phenomenon that the relevant information could appear in both direct and distant neighbors of matching concepts, the aggregated structural semantics of a concept should include not only its local neighbors, but also the related distant neighbors. In addition, to keep the matching performance, we use an attention mechanism to realize the semantic relatedness of different neighbors, which could further discover and aggregate important neighbors.

To address these issues, we propose a biomedical ontology matching framework, BioHAN, with a hybrid graph attention network. The underlying idea is to first enrich and refine the ontologies to be matched with the given axioms and auxiliary semantic descriptions from external resources, such as UMLS (Bodenreider, 2004). Then, the neighborhood information is aggregated within multiple hops in the enriched ontologies, capturing both local and global features, into hyperbolic representations that are complementary to each other. Both representations are jointly optimized to improve ontology matching performance. The main contributions of this study are listed as follows:

• We propose a matching method BioHAN for biomedical ontologies. BioHAN first enriches the ontologies for matching via the axioms and logical rules. Then it further learns the representations with the hierarchical structure to realize ontology matching.
• We propose a lightweight and effective way to enrich and refine ontology with the metadata, axioms, and auxiliary semantic information from external resources, which is helpful to discover and simplify the hidden and implicit semantics in ontologies.
• To capture the hierarchical features in an ontology, we leverage hyperbolic graph convolution layers to encode the parent and child concepts in the hyperbolic space.
• To further address the heterogeneity and better capture the semantics of concepts, we introduce an attention mechanism to weigh different neighbors and incorporate multi-hop neighbors to learn both the local and global hierarchical structures.
• We implement our proposed matching method and conduct systematic experiments on biomedical ontologies datasets. The evaluation of the Ontology Alignment Evaluation Initiative 2021 (OAEI 2021) shows that our method achieves significantly promising results.

The study is structured as follows. In Section 2, we describe relevant preliminaries of ontology matching and the overview of our proposed method. In Section 3, we illustrate the ontology-enriching operation, including ontology preprocessing and augmenting. In Section 4, the implementation details of our proposed matching method BioHAN are presented. Section 5 describes our experiments, the results, and the experimental analysis and discussion. In Section 6, related work about ontology matching is systematically reviewed and introduced. Section 7 summarizes our main findings, and presents perspectives on future work.

## 2 Preliminaries and Method Overview

### 2.1 Ontology Matching

Let C be the set of concepts, R be the set of relations, and T=C×R×C be the set of triples or statements, then a biomedical ontology can be represented as O=(C,R,T) . The matching between two ontologies *O*_*s*_ and *O*_*t*_ is $M=\left\{m_{k}|m_{k}\;=<\;\;e_{i},e_{j},r,s\;>\right\}$ (Euzenat and Shvaiko, 2007), where M is an alignment; *m*_*k*_ is a correspondence $<e_{i},e_{j},r,s>$ ; *e*_*i*_ and *e*_*j*_ are elements from *O*_*s*_ and *O*_*t*_, respectively; *r* is the semantic relation between *e*_*i*_ and *e*_*j*_; and *s* ∈ [0, 1] is the confidence about a correspondence. Therefore, an alignment M is a set of correspondences *m*_*k*_.

### 2.2 Graph Neural Networks

Graph neural networks (GNNs) are effective for various applications with graph-structured data (Zhou et al., 2020). A GNN framework usually has a graph encoder and a graph decoder, and its input is an adjacency matrix and features nodes and edges. The encoder uses the graph structure to propagate and aggregate information across nodes, and learns embeddings for local structure. A graph decoder is often used to compute similarity scores for all node pairs. Depending on the graph properties and aggregation strategies, some GNN frameworks have been proposed.

The vanilla GCN is a popular variant of the GNN (Kipf and Welling, 2016), in which the hidden representation of node *i* at the *l*-th (*l* > 0) layer $h_{i}^{\left(l\right)}$ is computed as

$$h_{i}^{\left(l\right)}=\sigma \left(\underset{j\in N_{i}\cup \left\{i\right\}}{\sum }\frac{1}{c_{ij}}W_{i}^{\left(l\right)}h_{i}^{\left(l-1\right)}\right)$$ (1)

where *σ*(⋅) is an activation function; ***W*** ^(*l*)^ is the weight matrix of the *l*-th layer and *c*_*ij*_ is for normalization; and $N_{i}$ denotes the neighbor set of node *i*. The vanilla GCN encodes node *i* as the mean pooling of the representations of its neighbors and node *i* itself from the last layer. The input vector fed to the first layer is denoted as $h_{i}^{\left(0\right)}$ .

A graph attention network (GAT) (Veličković et al., 2018) is a novel convolution-style neural network with masked self-attention layers. In contrast to the GCN, it allows for implicitly setting different weights to nodes of the same neighboring node. Moreover, analyzing the learned attention weights could improve interpretability. Formally, the attention weight $\alpha_{ij}^{\left(l\right)}\in R$ between *i* and *j* at the *l*-th layer is computed as follows:

$$\alpha_{ij}^{\left(l\right)}=\frac{\exp\left(LeaklyReLU\left(a^{T}\left[Wh_{i}\|Wh_{j}\right]\right)\right)}{\sum_{j\in N_{i}}\exp\left(LeaklyReLU\left(a^{T}\left[Wh_{i}\|Wh_{j}\right]\right)\right)}$$ (2)

Here, ⋅^*T*^ denotes transposition; ***a*** is an attention weight matrix; ‖ is the concatenation operation; and *LeaklyReLU* is used to achieve nonlinear transformation.

### 2.3 Method Overview

**FIGURE 3:**
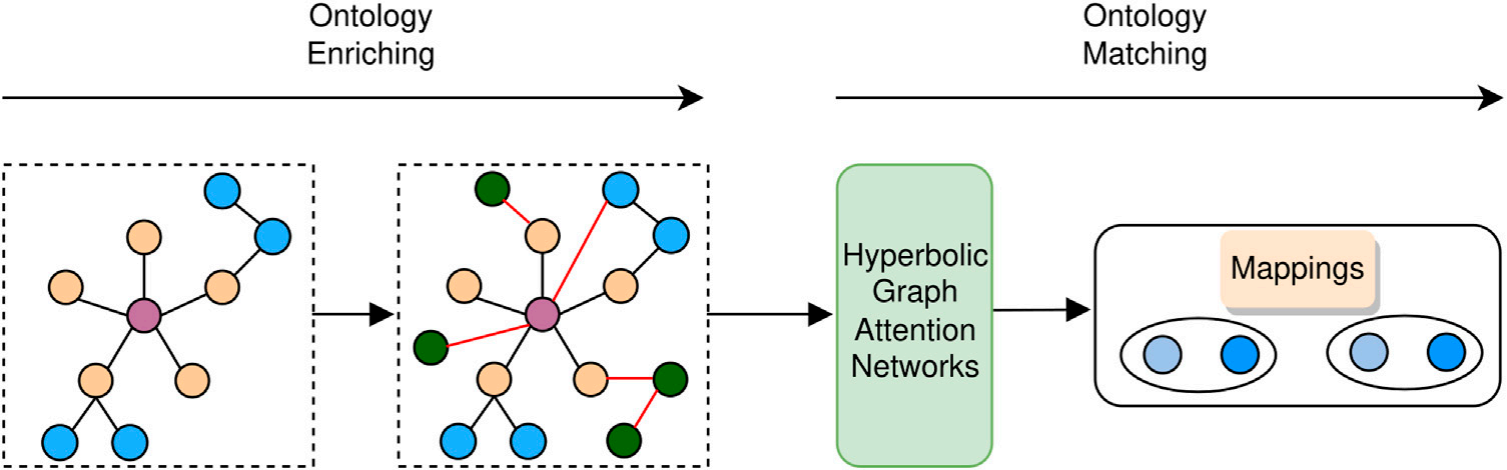
Framework of BioHAN.

As shown in Figure 3, our proposed BioHAN comprises two phases: ontology enriching and ontology matching. Given a biomedical ontology, the ontology enriching phase first preprocesses the ontology with the metadata and axioms, which complements the informative representations hidden in the ontology. It also explores matching seeds between the processed ontologies by supplementing some missing semantics through external resources. The ontology matching phase takes as input the derived ontology. Structures of ontologies are captured via graph attention networks for structural representation learning. Moreover, the lexical semantics of the concepts in ontologies are used, providing complementary clues for ontology matching.

## 3 Ontology Enriching

In this section, we will discuss ontology preprocessing and augmenting operation to enrich the initial ontology. Specifically, we first preprocess the ontology to discover the hidden semantics and represent them clearly. Then, we use ontology augmentation strategies to enrich the ontologies.

### 3.1 Ontology Preprocessing

We notice that there are two common facts in biomedical ontologies. On the one hand, some semantic information is hidden or unclear, which is expressed by complex axioms or ontology semantics. However, to further understand an ontology, such information is useful. On the other hand, some triples are used to describe the building and version information for an ontology. These statements simply increase the size of the ontology and are useless for the definitions of concepts and properties. Therefore, we conduct a preprocessing operation to refine ontologies. Specifically, we make the complex expressions of ontologies much simpler and clearer.

For the ontology language RDFS and OWL, they provide mechanisms for describing groups of related resources and the relationships between these resources, where OWL is an extension of RDFS, providing description logic-based primitives with richer expressive ability and stronger reasoning ability. In an ontology, containers (e.g., rdf:Bag, rdf:Seq, and rdf:Alt) and collections (e.g., rdf:List) are used to describe a set of resources in RDFS and OWL. They simplify the ontology expressions but hide some indirect semantics. We clearly define the semantics of the members in containers and collections, and then delete those redundant and complex statements. Table 1 shows the range of property “physical state” through a collection *rdf:List* in RDFS format. Through the RDFS description, we can know that for the property “physical state” in the ontology “http://bioontology.org/ontologies/fma,” its values could be one of “Gas,” “Liquid,” “Semi-solid,” and “Solid.” Each value is represented via *rdf:li*. However, the members would be represented as anonymous nodes while parsing the ontology, such as <physicalstate,range,BN> , <BN,range,Liquid> , where *BN* denotes an anonymous node with no specific meaning. These statements are difficult to understand directly. Therefore, it is necessary to formulate this implicit knowledge, such as <physicalstate,range,Liquid> .

**TABLE 1:** Example of ontology collection.

< rdf:Description rdf:about = “http://bioontology.org/ontologies/fma/physical state”
< rdfs:range >
< rdf:List >
< rdf:li > Gas < /rdf:li >
< rdf:li > Liquid < /rdf:li >
< rdf:li > Semi-solid < /rdf:li >
< rdf:li > Solid < /rdf:li >
< rdf:List >
< /rdfs:range >
< /rdf:Description >

In addition, to further mine the semantic descriptions in the biomedical ontologies, a rule-based reasoning method is proposed to discover the hidden information.

1) Enriching domain and range: given a property *p*_*a*_, let *p*_*b*_ be the sub-property of *p*_*a*_. Then we can infer that all semantics of the domain and range of *p*_*a*_ could be inherited by *p*_*b*_. According to this rule, the semantics of sub-properties will be defined more comprehensively.
2) Enriching the concept axioms: given a concept axiom (e.g. owl:oneOf, owl:intersectionOf, owl: unionOf, owl:equivalentClass, etc.), its equivalent semantics could be rewritten by following rules. If a complex concept *A* ⊓ *B* is defined by the axiom owl:intersectionOf, where the complex concept has a sub concept *C*, *A ⊐ C* and *B ⊐ C* could be added to the ontology. If one complex concept *A* ⊔ *B* is defined by the axiom owl:unionOf, where the complex concept has a super concept *C*, so *C ⊐ A* and *C ⊐ B* could be added to the ontology. Similarly, we can also rewrite semantics of owl:oneOf and owl:equivalentClass. Therefore, complex semantics of concept axioms could be clearly defined.
3) Enriching the property axioms: given a property axiom (e.g. owl:SymmetricProperty, owl: TransitiveProperty, owl:equivalentProperty, etc.), relevant semantic extension could be realized by following rules. If a property *p* is declared by axiom owl:SymmetricProperty and there is a statement <A,p,B> , a new statement <B,p,A> could be added to the ontology. If a property *p* is declared by axiom owl:TransitiveProperty and there are statements <A,p,B> and <B,p,C> , then a new statement <A,p,C> could be added to the ontology.
4) Enriching owl:sameAs axiom: given a statement <A,owl:sameAs,B> , then the equivalent individuals *A* and *B* could share their semantic information.
5) Enriching properties in the concept hierarchy: given <p,rdfs:domain,A> and <B,rdfs:subClassOf,A> , we can infer an implicit statement <p,rdfs:domain,B> . According to this rule, the property’s constraints about one concept could be extended to its sub-concepts.

### 3.2 Ontology Augmentation

Even though the derived ontologies have clearly specified the hidden semantics, they are still insufficient to some extent. Some semantic relationships are still missing, which may lead to the sparse problem of ontology structure. To alleviate this problem, we introduce several augmentation heuristics to enrich biomedical ontologies through the external domain resources, that is, UMLS.

#### 3.2.1 Concept Augmentation

We first explore the anchors between the ontologies to be matched and the external resources, which is performed by using a simple string-based technique. Then, for one concept in ontologies, the relative semantics (e.g. rdfs:label, owl:annotation, owl:equivalentClass, etc.) of its anchored concept in external resources could be transferred and added to the ontology. Concept augmentation can significantly enrich ontologies with available information from external resources.

#### 3.2.2 Neighborhood Augmentation

Relations between source and target concepts could also be derived from the anchored concepts in external resources. Specifically, if there is a relation between concepts *i* and *j* of the external resource, their anchors *i*′ and *j*′ are also linked by this relation. The goal is to reduce the semantic gap between ontologies by adding the missing structural information and solving the problem of sparse ontology graphs.

With the augmented ontologies, our matching framework enables sufficient learning of ontology representations. To match the concepts in ontology *O*_*s*_ and ontology *O*_*t*_, we use graph pooling to obtain the embeddings of concepts. After investigating different graph pooling methods (Hamilton et al., 2017; Ying et al., 2018), we choose mean-pooling to capture information across concept neighbors. Finally, the graph neural networks take the enriched ontologies *O*_*s*_ and *O*_*t*_ as input to find the alignments.

## 4 Matching Method

In this section, we first embed the elements in ontologies to low dimension vectors, and then discuss the hyperbolic graph attention mechanism. Subsequently, we elaborate on the matching computation and the model training in detail.

### 4.1 Embedding

The terminological descriptions of concepts within a biomedical ontology are generally represented by a sequence of words. We leverage deep learning-based embedding methods (Peters et al., 2018; Devlin et al., 2019) to derive a fixed-size terminological description embedding for each concept. In this study, we choose BioBERT, a high-quality medical language model pre-trained on PubMed abstracts and clinical notes (Lee et al., 2020), to encode concepts. Considering the domain specificity of biomedical ontology, the embedding models toward a specific task can provide significant benefits (Alsentzer et al., 2019; Peng et al., 2019), and are much more appropriate than the general pre-training language model. The embeddings are used as the initial states *h* ^0,*E*^ of concepts, where *E* indicates the low-dimensional vectors in the Euclidean space.

### 4.2 Hyperbolic Graph Attention

**FIGURE 4:**
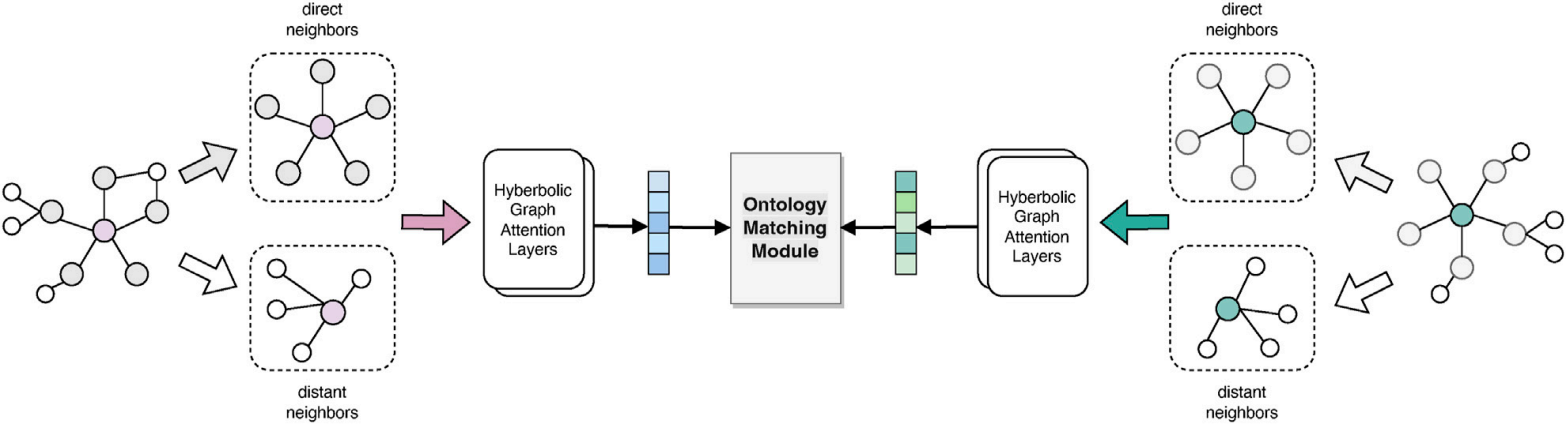
Hyperbolic graph attention layers in BioHAN.

Conventional GNNs typically capture the graph via message propagation to embed nodes into the Euclidean space. However, it could lead to the distortion of hierarchical structures (Nickel and Kiela, 2017). Hence, we transfer the node representations to a hyperbolic embedding space that can better capture the hierarchical characteristics of tree-like ontologies. In this study, we use a specific model, hyperbolic graph attention network (HGAT) (Zhang et al., 2021), which jointly implements both the expressiveness of a GAT and the superiority of hyperbolic geometry in capturing the hierarchical features. Moreover, multi-hop neighbors are also encoded into concepts, to comprehensively consider a broader context of concepts and alleviate the heterogeneity problem. The network architecture is shown in Figure 4.

#### 4.2.1 Hyperbolic Feature Projection

The hyperbolic graph attention layer first establishes transformation between the tangent (Euclidean) and Poincaré ball, which is carried out by exponential and logarithmic maps. Specifically, we project the vector in a tangent space to a hyperbolic manifold through the exponential map, whereas the logarithmic map reverses the hyperbolic representation back to the Euclidean space. The initial hyperbolic embedding $h_{i}^{0,H}$ of node *i* is

$$h_{i}^{0,H}=\exp_{o}^{K}\left(0,h_{i}^{0,E}\right)$$ (3)

where *K* determines the constant negative curvature −1/*K*(*K* > 0) and the tangent space is centered at point *o*. To transform the hyperbolic features from one layer to the next layer, we follow the following computation:

$$h_{i}^{l,H}=\left(W^{l}{⊗}^{K_{l-1}}h_{i}^{i-1,H}\right){⊕}^{K_{l-1}}b^{l}$$ (4)

where ⊗ and ⊕ are hyperboloid matrix multiplication and addition, respectively.

#### 4.2.2 Hyperbolic Attention Mechanism

To measure the importance of various neighbors and aggregate the neighbors’ features to the center node according to their semantic weights, a self-attention mechanism is performed on the nodes. To that end, one parameterized weight matrix ***W*** is applied to all nodes to conduct the shared linear transformation. Then, the attention coefficient can be represented with a self-attention weight ***a*** on the nodes as follows:

$$e_{ij}=a^{T}\left(W\left(h_{i}^{h},h_{i}^{h}\right)\right)$$ (5)

*e*_*ij*_ indicates the importance of node *j* to node *i*.

In addition, GAT considers only the local neighbors (i.e., one-hop neighbor nodes) for graph attention, while distant neighboring nodes can also contribute semantics to the central node. To reduce the effects of non-isomorphism in neighboring structures, we introduce distant neighboring information. Without loss of generality, we aggregate both the one-hop and two-hop neighboring information in ontologies, obtaining a proximity matrix.

$$P=\left(B_{1}+B_{2}\right)/2$$ (6)

where *B* is the transition matrix and *B*_*k*_ denotes the adjacency matrix of k-th hop. *B*_*ij*_ = 1/*d*_*i*_ if there exists an edge between *i* and *j* in the k-th hop, otherwise *B*_*ij*_ = 0. Then, *P*_*ij*_ denotes the topological weight that node *j* exerts on *i*.

To make coefficients comparable across different concepts, the attention weights are normalized via the softmax function.

$$\alpha_{ij}=softmax\left(e_{ij}\right)=\frac{\exp\left(e_{ij}\right)}{\sum_{k\in N_{i}}\exp\left(e_{ij}\right)}$$ (7)

Finally, using the topological weights *P* and applying the LeakyReLU nonlinearity, the coefficients can be expressed as

$$\alpha_{ij}^{\left(l\right)}=\frac{\exp\left(LeaklyReLU\left(P_{ij}\cdot a^{T}\left[W\vec{h_{i}}\|W\vec{h_{j}}\right]\right)\right)}{\sum_{k\in N_{i}}\exp\left(LeaklyReLU\left(P_{ij}\cdot a^{T}\left[Wh_{i}\|Wh_{j}\right]\right)\right)}$$ (8)

#### 4.2.3 Hyperbolic Attention-Based Aggregation

Similar to GAT, the hyperbolic graph convolution layer aggregates features from a node’s local neighbors. There is no notion of a vector space structure in a hyperbolic space, while the hyperboloidal aggregation requires multiplication by a weight matrix along with a bias operation. The main idea is to leverage the logarithmic projection to perform the Euclidean transformation and aggregation in the tangent space, and then transfer the obtained vectors back to the hyperbolic space. In addition, an attention mechanism is applied to learn the semantic relatedness between the neighboring nodes and the central node. Then, the neighbors’ features are assembled in accordance with the learned attention coefficients. The hyperbolic attention-based aggregation is defined as follows:

$$AGG^{K}{\left(h^{H}\right)}_{i}=\exp\left(\underset{j\in N_{i}}{\sum }\alpha_{ij}\log_{h_{i}^{H}}^{K}\left(h_{j}^{l-1,H}\right)\right)$$ (9)

To avoid semantic loss during the information propagation and maintain its transitivity between different convolutional layers, it is also necessary to incorporate the semantics of the central node itself.

$$AGG^{K}{\left(h^{H}\right)}_{i}=\exp\left(W_{AGG}\left(h_{i}^{H}+\underset{j\in N_{i}}{\sum }\alpha_{ij}\log_{h_{i}^{H}}^{K}\left(h_{j}^{l-1,H}\right)\right)\right)$$ (10)

where *W*_*AGG*_ is the aggregated weight matrix, and $h_{i}^{H}$ is the representation of the central node.

Finally, a non-linear activation function is used to increase the nonlinear expression ability and further improve the performance of the model. Specifically, BioHAN first applies Euclidean non-linear activation in the tangent space and then projects back to the hyperbolic space.

$$\sigma^{{⊕}^{K_{l-1},K_{l}}}\left(h^{H}\right)=\exp_{o}^{K_{l}}\left(\sigma \left(log_{o^{K_{l-1}}}\left(h^{H}\right)\right)\right)$$ (11)

The *l*-th layers of a hyperbolic graph attention layer are

$$h_{i}^{l,H}=\sigma^{{⊕}^{K_{l-1},K_{l}}}\left(AGG^{K_{l-1}}{\left(h^{l-1,H}\right)}_{i}\right)$$ (12)

where −1/*K*_*l*−1_ and −1/*K*_*l*_ are the hyperbolic curvatures at the (*l*-1)-th and *l*-th layer, respectively. After iterative propagation and update of representations between layers, the final hyperbolic vector representations ***h*** ^*H*^ can be obtained to represent the concepts.

### 4.3 Matching

Based on the learned concept representations ***h*** ^*H*^ from the hyperbolic graph attention layers, our matching module takes as input pairs of concept embeddings from *O*_*s*_ and *O*_*t*_, and then measures the semantic relatedness with a similarity metric function, defined as follows:

$$sim\left(c_{i},c_{j}\right)={\left\{\exp\left[\frac{1}{t}\left(d^{K}{\left(h_{i}^{H},h_{j}^{H}\right)}^{2}-r\right)\right]+1\right\}}^{-1}$$ (13)

where *d* ^*K*^ (⋅, ⋅) is the hyperbolic distance, and *r* and *t* are hyper-parameters. Then we iteratively match the concepts of two different ontologies using the Stable Marriage algorithm (SM) (Gale and Shapley, 1962) over the concepts’ pairwise similarities.

### 4.4 Training

To improve the matching performance of the proposed method, we jointly consider the reconstruction performance of the hyperbolic graph attention network and the matching performance of the matching module.

For the hyperbolic graph attention network module, the graph transition matrix of the final output should be as close as possible to the original graph structure. Therefore, the graph reconstruction loss should be minimized.

$$L^{H}=\underset{\left(i,j\right)\in E^{+}}{\sum }p\left(c_{i},c_{j}\right)+\underset{\left(i,j\right)\in E^{-}}{\sum }\omega \left[\mu -p\left(c_{i},c_{j}\right)\right]$$ (14)

where *E* ^+^ is the set of adjacency concept pairs; *E* ^−^ represents the corresponding negative samples; *μ* is the margin value; *ω* is a trade-off factor; and [⋅]_+_ = max (0, ⋅).

Then, for the matching module, we minimize the contrastive matching loss to actualize that the distances between pre-aligned concepts (positive) are as small as possible whereas the unmatched (negative) pairs have a relatively larger distance.

$$L^{M}=-\underset{\left(i,j\right)\in M^{+}}{\sum }logsim\left(c_{i},c_{j}\right)-\underset{\left(i,j\right)\in M^{-}}{\sum }\log\left(1-sim\left(c_{i},c_{j}\right)\right)$$ (15)

where $M^{+}$ is the set of seed correspondences between *O*_*s*_ and *O*_*t*_, and $M^{-}$ denotes the corresponding opposite ones.

The final joint loss function is defined as follows:

$$L=L^{H}+\alpha \cdot L^{M}$$ (16)

where *α* is positive hyper-parameters to control the trade-off among these loss components. The model is trained by minimizing the overall loss and optimized with an Adam (Kingma and Ba, 2014) optimizer.

## 5 Experiments

This section reports the experimental results. To verify the effectiveness of BioHAN, we used Python to implement our approaches in Pytorch and conduct the experiments on a computer with an Intel Xeon 4110 CPU, Nvidia 2080Ti GPU, and 64-GB memory.

### 5.1 Datasets

The experiments are performed on the biomedical evaluation benchmark from the Ontology Alignment Evaluation Initiative 2021 (OAEI 2021), which organizes annual evaluation campaigns aiming at evaluating ontology matching technologies. Biomedical ontologies are collected from the Large Biomedical track in OAEI 2021, including the Foundational Model of Anatomy Ontology (FMA), SNOMED CT, and the National Cancer Institute Thesaurus (NCI).

The FMA is an ontology for biomedical informatics that symbolically represents the phenotypic structure of the human body (Rosse and Mejino, 2003). FMA has 78,988 concepts together with 78,985 *isA* triples.

The NCI provides reference terminologies for clinical care, translational and basis research, public information, and administrative activities (Golbeck et al., 2003). It comprises 66,724 concepts and 59,794 *isA* triples.

SNOMED CT is a systematically organized collection of medical terms and provides comprehensive, multilingual clinical healthcare terminology for clinical documentation and reporting (Donnelly et al., 2006). It contains 1,22,222 concepts and 1,05,624 *isA* triples.

The matching tasks are FMA-NCI, FMA-SNOMED, and NCI-SNOMED. On account of the primary hierarchical architecture of ontologies and the deficiency of some other relations, except the hierarchical structure, we mainly consider the *SubClassOf* relationship of these datasets. In this study, we only focus on identifying one-to-one equivalence correspondences between concepts. Seed alignments are extracted from the UMLS (Bodenreider, 2004) and trained as positive samples. The negative alignments are sampled by randomly modifying one of the concepts in the positive sample pairs.

### 5.2 Evaluation Measures

We follow the standard evaluation criteria in OAEI 2021, calculating the precision (P), recall (R), and F1-measure (F1) for each matching task. Given a reference alignment set *Ref* and mapping correspondences *Map*, the precision and recall are calculated as follows:

$$P=\frac{\left|Map\cap Ref\right|}{\left|Map\right|}$$ (17)

$$R=\frac{\left|Map\cap Ref\right|}{\left|Ref\right|}$$ (18)

The F1-measure is the weighted harmonic average of precision and recall, defined as

$$F1=\frac{2\times P\times R}{P+R}$$ (19)

### 5.3 Experimental Settings

For our proposed BioHAN, each training takes 1,000 epochs with the learning rates among {0.01, 0.001, 0.0001}. The embedding dimension *d* is set to 128, and the initial input embedding has the size (d) 512. By default, we stack two hyperbolic graph attention layers in our model. For the hyperbolic graph attention decoder, we set *r* = 2.0, *t* = 1.0, and apply trainable curvature, which refer to the parameter configuration in MEDTO (Hao et al., 2021). We set the trade-off hyper-parameters *α*_1_ to 1.0. In addition, for each seed correspondence, we corrupt it and randomly replace it with five additional concepts to generate negative mapping pairs.

### 5.4 Experimental Results

#### 5.4.1 Ontology Matching Results

Table 2 shows the matching results of our proposed model compared with several matching methods or systems based on feature engineering and representation learning. The feature engineering-based top-performing matching systems are selected according to the results published in the Large Biomedical track by OAEI 2021. The comparative representation learning models are several recent typical embedding-based entity alignment models (MTransE, GCN-Align) and ontology matching models (DAEOM, MEDTO).

**TABLE 2:** Results of ontology matching.

Method	FMA-NCI			FMA-SNOMED			SNOMED-NCI		
	P	R	F1	P	R	F1	P	R	F1
AML	**0.958**	0.910	**0.933**	0.923	0.762	**0.835**	0.906	0.746	0.818
LogMap	0.940	0.898	0.919	**0.941**	0.689	0.796	**0.954**	0.667	0.785
LogMapBio	0.904	0.920	0.912	0.911	0.711	0.799	0.909	0.696	0.88
MTransE	0.627	0.640	0.633	0.505	0.475	0.490	0.254	0.378	0.304
GCN-align	0.813	0.783	0.798	0.763	0.729	0.746	0.745	0.775	0.760
DAEOM	0.882	0.689	0.774	0.719	0.693	0.706	0.891	0.682	0.773
MEDTO	0.944	0.874	0.908	0.871	0.762	0.813	0.901	**0.802**	0.849
BioHAN	0.930	**0.922**	0.926	0.898	**0.775**	0.832	0.911	0.797	**0.850**
BioHAN (w/o OB)	0.930	0.922	0.926	0.782	0.731	0.756	0.788	0.709	0.746
BioHAN (w/o HB)	0.831	0.822	0.826	0.771	0.729	0.749	0.850	0.711	0.774
BioHAN (w/o AM)	0.860	0.842	0.851	0.819	0.726	0.770	0.864	0.719	0.785
BioHAN (w/o MN)	0.893	0.849	0.870	0.822	0.745	0.782	0.877	0.701	0.779

Bold values represents the best results for the column in which they are located.

Compared with the extensively developed feature-based approaches such as AML, LogMap, and LogMapBio, our method achieves competitive results across all three tasks. The proposed BioHAN outperforms these rule-based approaches in measure R in FMA-NCI and FMA-SNOMED. AML, LogMap, and LogMapBio heavily rely on lexical features extracted from ontologies, while using representation learning could better capture some hidden semantics to discover more complex matching pairs. We can also observe that entity alignment models (MTransE, GCN-Align) designed for general knowledge bases are insufficient for domain-specific ontology matching. Compared to the representative matching methods (DAEOM, MEDTO), BioHAN also achieves competitive performance. The performance difference between MEDTO and BioHAN validates the importance of hierarchical features. BioHAN explicitly distinguishes and models the hierarchical structure, taking into account both the local and global hierarchical features, and obviously leads to promising results in biomedical ontology matching.

#### 5.4.2 Effectiveness of Ontology Enriching

To evaluate the effectiveness of the enriching phase, we further compare the *isA* triple size during ontology enriching. The detailed statistics concerning the size of each ontology matching task are shown in Table 3. Here, *Nodes* means the number of ontology entities, and *isA* is the edges between nodes with the relation *owl*: *subClassOf* in the ontology graph, while the *origin* and *enriching* represents the change in *isA* triple size before and after the enriching operation.

**TABLE 3:** Summary statistics of ontology enriching.

Ontology	Nodes	isA (origin)	isA (enriching)
FMA	78,988	78,985	78,985
NCI	66,724	59,794	75,454
SNOMED	1,22,222	1,05,624	2,03,942

We can observe that the change in the triple size of both the ontology NCI and SNOMED is explicit, while the FMA remains. The structure of NCI and SNOMED is sophisticated, and contains substantive *owl:intersectionOf* and *owl:unionOf* property links, especially SNOMED. Specifically, the *owl:intersectionOf* statement describes classes which contain precisely those individuals that are members of the class extension of all class descriptions in the list, while the *owl:unionOf* statement describes an anonymous class containing those individuals occurring in at least one of the class extensions in the list.

Moreover, we compare the matching performance between the proposed BioHAN and its variation BioHAN (w/o OB), which does not pay attention to ontology preprocessing and enriching. Results are also shown in Table 2. It is obvious that our model BioHAN consistently outperforms across these tasks, with an average increase of 6.0% in the F1 measure. This is attributed to the critically abundant structural features and implicit semantics added to ontology, which indicates that hierarchical information and implicit semantic descriptions contain considerably representative and critical features for ontology matching.

### 5.5 Discussion

#### 5.5.1 Impact of Ontology Enriching

According to the intuition that there are some hidden informative semantics in ontologies, especially for the complex one, we propose to enrich the ontology through ontology preprocessing and complementing. Through the statistics described in Table 3, numerous relationship descriptions are implicit but express a well-established role in ontology matching. Particularly in SNOMED, there are nearly more than twice the hierarchical relationships after enriching. Through the comparison of matching performance between BioHAN and BioHAN (w/o OB) shown in Table 2, we can draw the conclusion that the enriching phase indeed contributes to ontology matching with the sufficient complements of semantic and structural information.

#### 5.5.2 Performance Analysis of BioHAN

BioHAN uses the hyperbolic space projection to solve the intrinsical limitation in encoding complex patterns by its polynomial expanding capacity. In addition, it captures the structure of the concept by iteratively aggregating multi-hop neighborhoods with an attention mechanism. To gain an in-depth analysis of these components, we further design three variants of BioHAN: BioHAN (w/o HB), BioHAN (w/o AM), and BioHAN (w/o MN). BioHAN (w/o HB) replaces the hyperbolic projection with Euclidean space projection. BioHAN (w/o AM) removes the attention mechanism and regards all the neighboring nodes sharing the same weight. BioHAN (w/o MN) only considers the direct local neighbors and removes the multi-hop aggregation module in BioHAN. From the matching results reported in Table 2, we observe that the full model BioHAN achieves the best performance across all three matching tasks. It is also worth noting that both BioHAN (w/o AM) and BioHAN (w/o MN) perform better than BioHAN (w/o HB), which indicates that the hierarchical structure of the ontology captures much more essential and representative semantics. The hyperbolic graph convolutional layers can effectively encode such semantic information. By comparing the results of BioHAN (w/o AM) and BioHAN, it is obvious that the attention mechanism plays a significant role in solving the hierarchical heterogeneity of ontologies, which has improved the matching performance of 6.7% in F1 on average. For the multi-hop aggregation, by contrasting the performances of BioHAN (w/o MN) and BioHAN, it also exerts an important influence on capturing the semantics much more precisely than the complex hierarchical structures of biomedical ontologies. Multi-hop neighboring aggregation can discover much more complex matching pairs and has further improved the matching performance, especially in the measure R with an increase of 5.8% on average.

## 6 Related Work

### 6.1 Biomedical Ontology Matching

Traditional feature-based approaches have been investigated for ontology matching, using terminological, structural, and semantic features for the discovery of semantically similar elements. LogMap (Jiménez-Ruiz and Cuenca Grau, 2011) uses lexical and structural indexes to enhance its scalability. AML (Faria et al., 2013) also uses various informative features and domain-specific thesauri to complete ontology matching. Feature-based matching systems mainly rely on hand-crafted features to achieve specific tasks. Unfortunately, these methods will be limited for a given scenario with weak informativeness. Representation learning has an important impact on ontology matching. OntoEmma (Wang L et al., 2018) proposes a novel neural architecture for biomedical ontology matching, feeding into amounts of definitions and contexts to encode the concepts. It derives a variety of labeled data for supervised training and augments entities with complementary descriptions from external biomedical thesauri to improve the quality of alignments. MultiOM (Li et al., 2019) models features in ontologies from multiple views: lexical, structural, and resource. Then, it optimizes the vectors by limiting the sampling scope via structural relations in ontologies. Wang et al. (2021) systematically analyze and verify the effectiveness of multi-dimensions matching clues, subsequently aggregating the representation learning clues to boost biomedical ontology matching.

### 6.2 Graph Representation Learning

Recently, graph representation learning has gained great attention as graph neural networks (GNNs) have achieved state-of-the-art performance in various fields, such as community detection (Gargi et al., 2011), link prediction (Liben-Nowell and Kleinberg, 2007), graph alignment (Sun et al., 2018), and node classification (Bhagat et al., 2011). Some studies (Chen et al., 2017; Wang L et al., 2018) have used GNNs to achieve graph-embedded entity alignment, as similar entities often have similar neighborhoods in knowledge graphs (KG). Considering the attention mechanism, a graph attention network (Veličković et al., 2018) is proposed to learn the relatedness and importance propagated from the neighbors to the centered node. Then the neighboring message is incorporated with the measured weights. DAEOM (Wu et al., 2020) develops Siamese graph attention mechanism-based autoencoders to effectively integrate both the network structure and terminological description for deep latent representation learning in ontology matching. Recently, some researchers have substantiated that data in the form of graphs exhibit a non-Euclidean latent anatomy (Wilson et al., 2014; Bronstein et al., 2017). In addition, some recent works (Bronstein et al., 2017; Nickel and Kiela, 2017) have demonstrated the distinguished representation ability of hyperbolic manifold to model datasets with hierarchical layouts, as the hyperbolic geometry performs well in reflecting hierarchical representations naturally. Inspired by this insight, numerous research studies focus on investigating the hyperbolic geometric graph models, such as those by Nickel and Kiela (2017); Nickel and Kiela (2018); Ganea et al. (2018); and Hao et al. (2021). MEDTO (Hao et al., 2021) encodes the hierarchical features of concepts through hyperbolic graph convolution layers and further captures both local and global structural information of concepts via heterogeneous graph layers to learn better concept representations for ontology matching, and has achieved remarkably competitive performance.

## 7 Conclusion

In this study, we propose BioHAN for biomedical ontology matching, a hybrid graph neural network-based auto encoder to effectively integrate hierarchical structures for latent representation learning in biomedical ontology matching. The proposed framework BioHAN executes ontology enriching to refine and complement the semantic information and hierarchical structures. Then it encodes the geometrical properties of concepts into a hyperbolic space to capture the hierarchical information through hyperbolic graph attention layers. We further implement multi-hop neighboring aggregation to incorporate both the local and global hierarchical structures with an attention mechanism to learn better concept representations for ontology matching. Our experiments conducted on a variety of biomedical ontologies demonstrate the effectiveness of BioHAN. Nonetheless, our approach only considers the *subClassOf* relationship in the ontology, which would restrict the capability of graph representation learning. In the future, it is promising to investigate some other types of non-isomorphism relations and incorporate the heterogeneous features into biomedical ontology matching. In addition, as for the large-scale biomedical ontology, the matching efficiency would also be taken into account in future research.

## Data Availability

The original contributions presented in the study are included in the article/Supplementary Material; further inquiries can be directed to the corresponding author.
